# At risk of reproductive disadvantage? Exploring fertility awareness among migrant women in Germany

**DOI:** 10.1016/j.rbms.2021.11.007

**Published:** 2021-12-31

**Authors:** Nadja Milewski, Sonja Haug

**Affiliations:** a Federal Institute for Population Research, Wiesbaden, Germany; b Eastern Bavarian Technical University, Regensburg, Germany

**Keywords:** Fertility knowledge, Migrant assimilation, Minority groups, Europe, Age-related fertility decline, Non-patient sample

## Abstract

This study examined awareness about fertility among immigrant women and non-migrants in Germany. The social relevance of infertility and fertility treatment is increasing in Western countries due to continually low overall birth rates, a high rate of childlessness, and a gap between the desired and actual numbers of children. While there is growing interest in infertility and reproductive medicine in general, previous studies have rarely included immigrant or ethnic minorities in Europe. This study investigated whether knowledge on the age-related fertility decline (ARFD) varies between migrant groups and the majority group, and the role of education. Working hypotheses were drawn from theoretical considerations on frameworks of migrant assimilation. The analysis was based on data collected in a social science pilot study on reproductive medicine, representative of the general population (‘NeWiRe’ 2014–2015). The sample included 962 women aged 18–50 years living in Germany. Approximately 81% of the sample were immigrants who originated from Turkey, Poland, the Balkan countries or countries of the (post-Soviet) Commonwealth of Independent States. While rather poor overall, knowledge on ARFD was found to be significantly lower in the migrant groups compared with the majority group. This minority-group disadvantage cannot be explained by sociodemographic or cultural variables. Future research should include minority groups in empirical studies on awareness about fertility in order to better understand the causes of this disadvantage, and the potential reproductive needs of migrants.

## Introduction

While there is growing interest in infertility in general – as well as in the attitudes, demand for, and use of assisted reproductive technology (ART) – previous studies on these topics in Europe have rarely examined immigrant groups or ethnic minorities in particular. This study aimed to fill this gap and investigated awareness about fertility among immigrant women and non-migrants in Germany. This paper is a part of the Symposium Issue ‘Making families through assisted reproductive technologies: causes, experiences and consequences in international context’ (guest edited by Anne-Kristin Kuhnt and Jasmin Passet-Wittig).

The aims of this study were three-fold. First, factual fertility knowledge is essential in modern family planning. Current demographic developments in industrialized countries show that women, men and couples postpone their childbearing to older ages, with significant percentages of populations remaining childless or having fewer children than desired (Schmidt et al., 2012, Sobotka and Beaujouan, 2018). Two recent literature reviews demonstrated that, around the globe, adults of reproductive age are not sufficiently informed about fertility, pregnancy and reproductive medicine (Garcia et al., 2018, Pedro et al., 2018). In particular, knowledge about age-related fertility decline (ARFD) is crucial in fertility intent because fertility decreases with age due to a reduction in both the quantity and quality of gametes (Baird et al., 2005). Advanced maternal age is a non-modifiable risk factor for reduced fecundity and infertility, and it decreases the likelihood of natural conception (Dunson et al., 2002, Evers, 2002). Due to its effect on gamete production, advanced age also reduces the success rate of any ART treatment (Habbema et al., 2015). This study contributes to the growing literature on awareness about fertility – a concept defined as the understanding of fertility and fecundity as well as related risk factors; this also includes awareness about societal and cultural factors affecting family planning and family building needs (Zegers-Hochschild et al., 2017).

Second, this study focused on migrant women in Europe. Immigrant fertility has received growing scholarly attention in Western European countries over the past two decades, especially with regard to migrants from the Global South. Aspects of reproductive health and family planning among migrant populations, however, are underinvestigated areas in fertility research (Milewski and Mussino, 2018). Large-scale quantitative studies on (in)fertility and reproductive health in Europe and elsewhere have mainly focused on the majority populations, and neglected subpopulations such as migrant or ethnic minorities. Such a discrepancy in scholarly interest may be attributed to an overall societal concern with a perception of ‘hyper-fertility’ of women from non-Western countries, as reported nearly two decades ago (Inhorn and van Balen, 2002). Primarily US-based scholars have drawn attention to this problem of under-representation of minority groups (Inhorn, 2020). In Europe, however, few research projects have focused explicitly on ‘marginalized reproduction’ (quoting the book title by Culley et al., 2009a) and addressed ethnic-cultural diversity in patients for reproductive medicine.

Third, while the main objective of this study was to include women of migrant groups in research on infertility and reproductive medicine, the authors also aimed to extend the literature by studying women from the general population (i.e. a non-patient and non-professional sample). This is important in order to better understand fertility decision-making processes and the potential causes of infertility. Patient studies include selected groups (i.e. those who seek treatment, thereby excluding those who do not). The selected groups may have higher socio-economic status and may be more aware of reproductive problems; their fertility knowledge may therefore appear better than that of the general population (Bunting et al., 2013, Greil, 1997).

These three motivations were brought together by asking whether migrant groups may be at risk of experiencing reproductive disadvantage, and studying their knowledge on ARFD. The country context of this study was Germany, the leading destination in Western Europe for several decades. The country has accommodated immigrants who came for various reasons and from a variety of regions of origin since the end of World War II in 1945. The proportion of immigrants, including subsequent generations, has been rising steadily and today represents approximately one-quarter of the population. This study focused on women of several migrant groups and compared their knowledge with that of non-migrant women. Moreover, the role of education in the differences between these groups was examined. Data collected in a social science pilot study on ART (‘NeWiRe’ 2014–2015; Haug et al., 2018), representative of the general population, were used. This study oversampled immigrants who originated from Turkey, Poland, the Balkan countries or countries of the (post-Soviet) Commonwealth of Independent States (CIS). Germany is an interesting setting for such a case study, not only because of its increasingly multi-ethnic context. Its persistent below-replacement fertility and fertility differentials between minority and majority groups are also relevant for the topic of this Special Issue ‘Making families through assisted reproductive technologies: causes, experiences and consequences in international context’. Germany’s rate of childlessness exceeds 20% and is one of the highest in Europe (Sobotka, 2017).

## Theoretical background

### Determinants of fertility knowledge

There is growing literature on awareness about fertility in general, and on ARFD and ART in particular, which typically focuses on industrialized countries. Garcia et al. (2018) and Pedro et al. (2018) noted in their review papers that some studies lacked quality (for different reasons), that findings were inconsistent, and that results were not fully comparable. Despite these limitations, some results can be generalized: awareness about fertility has been shown to be rather low. This inadequate knowledge concerns all aspects of reproductive health and fertility, including ARFD, ‘myths’ about conception, the fertile period, and risk factors for infertility other than age.

On the individual determinants of fertility knowledge, past work has found some variation by sociodemographic characteristics, although results are partially contradictory: women, individuals with higher levels of education, people having difficulties with conceiving, and those who planned their pregnancies have greater knowledge about fertility. Previous studies found no impact of having or desiring to have children, while age was inconsistently associated with awareness about fertility (Garcia et al., 2018, Pedro et al., 2018). At macro level, international comparisons revealed some variation by country, with people living in highly-developed countries having better fertility knowledge than women and men in so-called ‘developing countries’ (Bunting et al., 2013). It is noteworthy that few analyses have included countries in the Global South. This lack of scholarly attention may reflect the perception that countries with more ‘traditional’ demographic behaviour (i.e. early and rather high fertility) may not be at risk of delayed childbearing and infertility (Garcia et al., 2018).

### Empirical findings on minority groups

Knowledge about geographical variation in fertility and awareness about fertility is important in order to understand any differences between international migrants and members of the populations of the respective destination countries. Remarkably, in the extensive literature reviews undertaken by Garcia et al. (2018) and Pedro et al. (2018), which included over 40 and 70 international publications, respectively, not even a handful of studies focused explicitly on migrant status or ethnicity, or included them as determinants of awareness about fertility. Their conclusions therefore refer implicitly to majority populations alone, with no comments for any minority group in any regional context, be it with respect to race or ethnicity, migrant status or religious diversity. Overall, migrant groups appear to be understudied – and thus, marginalized – in research on awareness about fertility.

In research on migrant fertility in Europe, it remains an open question whether a certain immigrant group in a certain country develops into a minority group (Kulu et al., 2019). Minority groups and immigrant groups may overlap or be distinct from each other, or a group of migrants may develop gradually into a minority group over time and generations (Coleman, 1994, Haug, 2000). Bean and Tienda (1990) listed four criteria that characterize minority groups: (i) each of the subgroups constitutes only a small proportion of the total population of a country; (ii) members of the particular group experience a sense of self-awareness as belonging to the group as its members; (iii) members of the particular group experience a degree of discrimination by members of the majority group; and (iv) the members of the particular group are, to some extent, discernible in their appearance as its members.

Migrant groups in Europe and ethnic minorities in North America may be constituted by different social indicators and historical developments. They share, however, similarities with respect to their below-average socio-economic achievements, occupational hazards, environmental risks and poorer housing conditions, experiences of discrimination and lifestyle factors, which are associated with poorer health outcomes as well as demographic behaviour (Bean and Tienda, 1990, Coleman, 1994, Foner and Alba, 2008). On the one hand, these factors contribute to a higher risk of infertility in minority groups, while at the same time, minority groups are less likely to seek and receive treatment (Greil et al., 2011, Inhorn et al., 2009). On the other hand, these social factors are conducive for higher average fertility in minority groups and lower rates of childlessness. Migrant fertility in Europe seems to be highest among first-generation migrants, and declines in subsequent generations along with social and structural assimilation processes (Kulu et al., 2019, Milewski, 2010), in line with classical migrant assimilation theory (Gordon, 1964).

Very little is known about fertility knowledge in minority groups. Based on the few existing studies, it appears that awareness about fertility is consistently lower in minority groups than in majority groups (David et al., 2000, Deatsman et al., 2016, Swift and Liu, 2014). Previous work has not elaborated on the knowledge gap by ethnicity other than noting that the gap found was ‘somewhat unexpected’ (Deatsman et al., 2016: 102). David et al. (2000) related lower knowledge to lower levels of education and gainful employment of patient migrants (in Germany).

In the demographic contexts of highly industrialized countries with relatively low levels of fertility, research is dominated by the rational-choice motivated concept of family planning and conscious fertility-decision making, which appears to be the ideal of (White, Christian) middle-class families. Some research suggests that members of migrant groups from collectivistic societies may be less likely to share these motivations (Greil et al., 2011, Milewski and Mussino, 2018). The literature on migrant and ethnic minorities in North America and Europe shows that family formation and fertility are characterized by traits of an absence of conventional planning, such as younger ages at parenthood and larger overall numbers of children. At the same time, persistent differences in gender role attitudes emphasize motherhood in minority groups originating from Muslim or South Asian countries more than they do in majority populations, with ‘an almost obligatory procreation mandate’ [e.g. for Turks in the UK (Gürtin-Broadbent, 2009)], and childlessness carrying a social stigma [e.g. in certain ethnic groups in the USA (Culley and Hudson, 2009, Greil et al., 2011)].

### Working hypotheses

This background section will conclude by formulating the working hypotheses for the empirical analyses. The migrant women in this study were from Turkey, Poland, and successor states of the Soviet and Yugoslav federations. These regions have undergone substantial demographic change in the past decades, and vary in their fertility rates. Compared with Germany, they have low rates of childlessness and rather early entrance into motherhood (Sobotka, 2017). Fertility knowledge in these countries is generally poorer than in Germany (Bunting et al., 2013). The first working hypothesis concerns the influence of the migrants’ country of origin. Literature is lacking on the ideational or cultural dimension of assimilation among immigrants in Germany or in other European countries, with minorities from Muslim countries being an exception. Most literature on attitudes and values among women from countries with a Muslim tradition indicates that it is mainly first-generation immigrants who are likely to maintain the attitudes and values dominant in their country of origin during their socialization phase abroad (Milewski and Mussino, 2018). These findings are in line with Almond and Verba’s (1989) theory of political socialization through school, work or the media according to which societal climate (e.g. what is created by public discourse, policies and their implementation) is reflected in public attitudes. In the first working hypothesis, it is assumed that ARFD knowledge is lower among migrants than in the majority group due to fertility knowledge differentials between the respective countries of origin and Germany.

The second working hypothesis concerns the role of cultural assimilation across migrant generations and the role of socio-economics. Classical assimilation theory (Gordon, 1964) would propose that subsequent migrant generations gradually adapt to the destination society. The second generation has been described as being between two cultural reference points, particularly when the cultural difference between their parents’ origin and the destination country is rather large, as in the case of Muslims migrating to Europe. On the one hand, descendants of immigrants may be influenced by channels of political socialization in the destination country, and the institutional context for larger parts of their life course. Thus, their fertility knowledge may resemble that of non-migrants rather than that of their parents. On the other hand, additional factors may also play a role in the descendants’ knowledge, such as intergenerational transmission of the parents’ culture and knowledge. Some qualitative work suggests that migrant communities may draw on a range of different knowledge sources related to fertility and infertility, as well as its causes and treatments, in addition to the Western medical model of fertility (Culley and Hudson, 2009). In the second hypothesis, it is expected that knowledge differentials will diminish in the second generation due to assimilation processes. Moreover, any differentials may decrease when controlling for the mediating role of sociodemographic compositions of the groups, as well as for cultural variables referring to gender roles and religiosity (Foner and Alba, 2008).

Third, the role of social stratification will be examined in greater detail by studying educational groups separately. Colen (1986) introduced the concept of stratified reproduction to compare social groups; this has been applied mainly to abortion, sterilization, birth control and, to a small extent, infertility (Culley et al., 2009a, Greil et al., 2011). Research on patients provides ample evidence for below-average treatment rates of Black and Latinx people in North America (Davis, 2020, Feinberg et al., 2006, Greil et al., 2011, Shapiro et al., 2017, Stephen and Chandra, 2000, Wiltshire et al., 2019). If migrants have a disadvantage mainly due to their socio-economic risk factors, differentials should be smaller or non-existent among highly educated migrants.

## Empirical material and methods

### Data

The data used in this study were gathered in 2014–2015 in a research project on the role of social networks and knowledge transfer regarding assisted reproductive medicine (‘NeWiRe’; Haug et al., 2018), carried out by a project team at the Eastern Bavarian Technical University in Regensburg. Some parts of the questionnaire for this data collection replicated a German survey dating from 2003 (Stöbel-Richter et al., 2009). The indicator for ARFD was taken from this survey. Unlike Stöbel-Richter et al.’s project, one specific goal of the NeWiRe survey was to include, in addition to German native-born women, first- and second-generation immigrants. In this endeavour, the NeWiRe project had the character of a pilot study. Its target groups were the four largest immigrant groups living in Germany, based on the ‘migrant background’ criteria used in official statistics and in the German microcensus. These were migrants from Turkey and Poland, as well as migrants from nine states belonging to the (post-Soviet) CIS countries and seven Balkan countries. The statistical criteria ‘migrant background’, as used in official statistics in Germany, is defined as having foreign citizenship, own migration experience or at least one parent who immigrated to Germany (Destatis, 2018). The sample for the NeWiRe survey was drawn from the nationwide telephone register in Germany, using disproportionally layered random sampling by means of an onomastic (name-based) procedure with the aim of having a representative sample of the respective migrant groups. The onomastic method worked rather well in the groups from Poland, Turkey and the CIS countries, with correct identification rates ranging from 86% to 95%; however, correct identification was rather modest for individuals originating from Balkan countries (61%) (Haug et al., 2018). As a result of this sampling method, 1001 interviews were conducted. The data were collected via a standardized telephone survey (CATI) of women living in Germany aged 18–50 years. The questionnaire was available in German, Turkish, Polish, Russian and Serbo-Croatian; the interviews were carried out by bilingual interviewers in order to allow respondents to switch between German and their respective mother tongue or second language at any point in the interview.

The final sample used in this analysis consisted of 962 women (39 respondents were excluded from the analyses due to missing or unreliable information on their migrant background). Approximately 81% of the women in the study sample belonged to a migrant group and 19% were non-migrants. Among the immigrants, generations were distinguished by the respondents’ country of birth. Approximately two-thirds of them had migrated to Germany themselves or with their parents (mean length of stay was 24 years), while the remaining one-third of women were born in Germany.

### Dependent variable and method

This analysis centres on ARFD knowledge. The question ‘At what age do you think female fertility starts to gradually decline?’ was used. Possible answers were given in age groups, of which the categories ‘from age 25 onwards’ and ‘from age 30 onwards’ can be interpreted as ‘correct’ answers. Garcia et al. (2018) provided a detailed overview of several ways to ask for ARFD and to estimate the (correct) results. Many surveys differentiate in their questionnaire between a ‘slight decline’ (i.e. starting at 25 years of age) and a ‘marked decline’ (i.e. at 35 years of age) in fertility. As the question used in the present study asked for the ‘start of a gradual decline’, age groups corresponding to a ‘slight decline’ were also considered to be correct (Hammarberg et al., 2013, Stoebel-Richter et al., 2012, Vassard et al., 2016). ‘Incorrect’ answers were all age groups from 35 years onwards, as well as ‘during menopause’ and ‘don’t know’.

The empirical analyses were commenced by displaying a bivariate description of all answer categories on the ‘knowledge’ question by the respondents’ (or their parents’) country of origin; bivariate test statistics were based on Chi-squared tests for nominal variables and *t*-tests for metric variables. Multivariate binary logistic regression analyses were performed, where ‘correct knowledge’ was the dependant variable (1 = ‘correct’, 0 = ‘incorrect’ answers). Independent variables were added on a stepwise basis. Results have been displayed as average marginal effects (Mood, 2010). The analyses were undertaken using Stata Version 14 (Stata Corp., College Station, TX, USA).

### Explanatory variables and sample

The main explanatory variable was belonging to one of the migrant groups or the majority group; this considered the country or region of origin (=birth) of the respondents and their parents, and distinguished between first- and second-generation migrants among women from Turkey and the Balkan countries, as well as German non-migrants. Three sociocultural variables were used, which may mediate the relationship between belonging to an ethnic group and ‘knowledge’. First, the level of educational attainment was measured by the highest school degree obtained: none/lower secondary education (corresponding to ‘Hauptschule’), intermediate secondary education (corresponding to ‘Realschule’ or ‘Mittlere Reife’), and upper secondary education (corresponding to ‘Abitur’ or ‘Fachhochschulreife’). Second, a variable for individual religious behaviour related to family planning was used, by querying the degree of agreement with a statement on family planning methods: ‘I practise family planning in accordance with religious rules.’ Third, an indicator for gender role attitudes was used. The respondents were asked how much they agreed with the statement, ‘A woman needs to have children to live a fulfilled life.’ The answers to the questions on religious family planning and gender role attitudes were scored using a five-point Likert scale, which was dichotomized [1 = the respondent agreed (completely) with the statement; 0 = the respondent disagreed (completely) with the statement or chose ‘neither nor’ for an answer]. Further independent variables were used as controls in the multivariate analysis in order to account for sociodemographic differences between the groups under study. Age was used as a metric variable. The variable ‘union status’ measured whether or not the respondents were married, were living with a partner but not married, or did not have a partner at the time of the interview. A parenthood variable indicated whether or not the respondents had children (0, 1–2, or ≥3), and another variable accounted for whether or not the respondents said that they wanted to have a child/another child. In addition, whether or not the respondents had undergone any ART treatment prior to the survey was considered.

Table 1 gives a descriptive overview of the sample by the main categories. Approximately 19% of the sample were German non-migrants, 26% of the women were from CIS countries, 20% were from Poland, 20% were from Turkey, and 16% were from a country in the Balkan region. Considering country of origin and migrant generation simultaneously, the proportion of second-generation migrants was approximately 2% in women from the CIS countries, 10% in women from Poland, 32% among women from Turkey, and 27% among women from the Balkan countries. In migrant groups coming from Turkey and the Balkan countries, it was possible to distinguish between first- and second-generation migrants; however, due to the small sample size of second-generation migrants in women from the CIS countries and Poland, no distinction could be made in the multivariate analyses. Regarding their religious affiliations, the women from Poland and Turkey were dominated by a single religious affiliation; >90% of Polish women belonged to the Roman Catholic Church and >90% of the Turkish women indicated a Muslim affiliation. Women from the Balkan countries, CIS countries and Germany reported two significant religious denominations, and a high proportion of women from these countries reported that they were not religious. As the variable ‘religious affiliation’ correlates with the country of origin, this indicator was not used in the multivariate analyses, although it shows the religious diversity of the sample. The differences in sociodemographic characteristics between the migrant groups and the non-migrant majority population appear similar to the results of previous studies (Table 1).

**Table 1:** Description of the sample, by origin group (%).

				*Migrants by origin*			
	Total	Germany	Migrant total	CIS countries	Poland	Turkey	Balkan countries
*Migrant generation*							
First generation	na	na	84.1	98.4	89.9	67.6	73.5
Second generation			15.9	1.6	10.1	32.4	26.5
*Age (mean)*	38.0	38.8	37.8	38.5	38.0	36.4	38.3
Union status***							
Single (not married/divorced/widowed)	13.0	14.8	12.6	9.1	10.6	18.6	13.2
Non-marital cohabitation	11.5	20.9	9.4	9.5	16.9	3.7	6.6
Married	75.5	64.3	78.1	81.3	72.5	77.7	80.1
Parenthood***							
Childless	17.3	25.8	15.3	8.7	20.6	20.2	13.2
1–2 children	56.3	53.8	56.9	65.9	59.3	42.6	57.0
3+ children	26.4	20.3	27.8	25.4	20.1	37.2	29.8
*Wish to have (another) child*							
No	69.0	69.2	69.0	69.0	66.7	69.1	71.5
Yes/not decided yet	31.0	30.8	31.0	31.0	33.3	30.9	28.5
*ART treatment experience*							
Yes	7.3	8.2	7.1	6.0	8.5	6.4	7.9
No (incl. mv)	92.7	91.8	92.9	94.0	91.5	93.6	92.1
School education***							
None/primary/lower secondary	15.5	9.3	16.9	10.3	4.8	35.1	20.5
Intermediate secondary	41.9	42.3	41.8	57.5	31.7	33.0	39.1
Upper secondary	39.3	47.3	37.4	30.2	56.6	27.1	38.4
mv	3.3	1.1	3.8	2.0	6.9	4.8	2.0
Family planning conforming to religious regulations***							
‘Completely disagree’	27.8	53.8	21.7	34.1	14.3	5.3	30.5
‘Disagree’	18.6	21.4	17.9	26.2	22.2	8.5	10.6
‘Neither nor’	10.3	11.0	10.1	12.7	9.5	3.2	15.2
‘Agree’	21.0	8.2	24.0	16.7	30.7	27.1	23.8
‘Completely agree’	21.0	3.8	25.0	9.5	21.7	54.3	18.5
mv	1.4	1.6	1.3	0.8	1.6	1.6	1.3
‘*A woman needs children…*’ ***							
‘Completely disagree’	7.7	17.6	5.4	2.4	8.5	4.3	7.9
‘Disagree’	10.1	13.7	9.2	8.7	12.2	7.4	8.6
‘Neither nor’	11.1	19.8	9.1	8.3	12.2	5.9	10.6
‘Agree’	25.2	28.0	24.5	21.0	23.3	27.7	27.8
‘Completely agree’	45.4	20.9	51.2	58.7	43.4	53.7	45.0
mv	0.5	0.0	0.6	0.8	0.5	1.1	0.0

*n*	*962*	*182*	*780*	*252*	*189*	*188*	*151*
*% at total sample*	*100*	*18.9*	*81.1*	*26.2*	*19.6*	*19.5*	*15.7*

*Source: NeWiRe survey 2014/2015. n* = 962*.*

*Note: Significance for bivariate association between variable and origin group (chi^2^ or t-test);* *P ≤ .05; **P ≤ .01; ***P ≤ .001.

*CIS: Armenia, Belarus, Kazakhstan, Kyrgyzstan, Russian Federation, Tajikistan, Turkmenistan, Uzbekistan, Ukraine;*

*Balkan countries: Bosnia and Herzegovina, Croatia, Kosovo, North Macedonia, Montenegro, Serbia, Albania.*

*mv = missing values, na = not applicable.*

## Results

### Bivariate findings

**Fig. 1:**
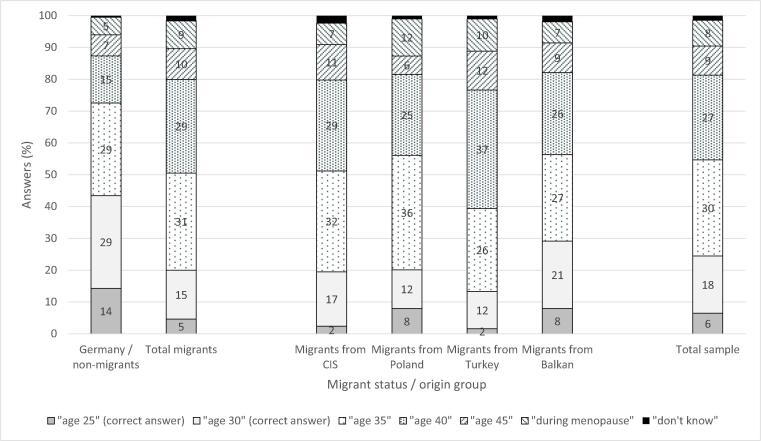
Knowledge of age-related fertility decline by origin group (%). Respondents were asked: ‘At what age do you think female fertility starts to gradually decline?’ Source: NeWiRe survey 2014–2015 (*n* = 962).

Fig. 1 shows the bivariate overview of respondents’ answers regarding female fertility decline. Two main results emerged: at only 43%, the percentage of ‘correct answers’ (i.e. corresponding to a ‘slight decline’) in non-migrant German women was rather modest. Large differences were evident between the majority and migrant groups. Between 14% (Turkey) and 29% (Balkan countries) of the respondents in each of the four groups gave correct answers regarding ARFD. It is also worth examining the answers rather far from being ‘correct’ (i.e. answers indicating false or no knowledge): approximately 45% of all respondents said that they did not know the answer or that fertility starts declining at ‘age 40′ or ‘during menopause’, with similar patterns in group differences as in the ‘correct’ answers.

### Multivariate models by origin group

Table 2 shows the results of the multivariate analyses. These models distinguished between migrant generations among women from Turkey and from the Balkan region. The pattern of significant differences in knowledge about ARFD between non-migrants and almost all migrant groups seen in the bivariate statistics (Fig. 1) remained, with second-generation migrants from Balkan countries being the exception. Being a first-generation migrant from Turkey decreased the probability of the respondent answering the question on ARFD correctly by 0.32 percentage points (Model 0). Model 1 also included age, children and union status as indicators of the sociodemographic composition of the groups in the sample, as well as using the desire to have a child/another child and previous ART treatment experience as controls. The only control variable associated with slightly significant differences in knowledge on ARFD was whether the respondents said that they had not yet finished their childbearing phase. However, these variables did not explain the significant differences found between non-migrants and second-generation migrants from Balkan countries on one hand, and other migrant groups on the other hand.

**Table 2:** Multivariate results on correct knowledge about age-related fertility decline.

	**Model 0**		**Model 1**		**Model 2**		**Model 3**	
*Origin group (Ref = Germany)*								
Balkan countries/first generation	−0.02	***	−0.19	**	−0.18	**	−0.12	*
Balkan countries/second generation	0.02		0.00		0.00		0.02	
Turkey/first generation	−0.32	***	−0.30	***	−0.28	***	−0.20	**
Turkey/second generation	−0.27	***	−0.28	***	−0.28	***	−0.21	**
Poland	−0.23	***	−0.23	***	−0.25	***	−0.19	***
CIS countries	−0.24	***	−0.24	***	−0.23	***	−0.19	***
*Age*			0.00		0.00		0.00	
*Union status (Ref = married)*								
*Non-marital cohabitation*			0.06		0.05		0.04	
Single and no cohabitation (not married/divorced/widowed)			−0.02		−0.01		−0.02	
*Parenthood (Ref = 1*–*2 children)*								
None			−0.01		−0.03		−0.04	
3 + children			0.02		0.03		0.04	
*Wish to have (more) children (Ref = no)*								
Yes/not decided yet			0.06	°	0.06	°	0.08	*
*ART treatment experience (Ref = no)*								
Yes			0.06		0.06		0.06	
*School education (Ref = none/primary/lower secondary)*								
Intermediate secondary					0.03		0.03	
Upper secondary					0.11	**	0.09	*
*Family planning conforming to religious regulations (Ref=*‘*(completely) disagree*’*/*‘*neither nor*’*)*								
‘(Completely) agree’							−0.11	***
‘*A woman needs children…*’ *** *(Ref=*‘*(completely) disagree*’*/*‘*neither nor*’*)*								
‘(Completely) agree’							−0.03	
*R^2^ (Cragg & Uhler's)*	*0.09*		*0.11*		*0.12*		*0.14*	

*Source: NeWiRe survey 2014/2015. n = 962.*

*Note: Binary logistic regression, dependent variable: 1 = correct, 0 = not correct;* °*P ≤ .1;* *P ≤ .05; **P ≤ .01; ***P ≤ .001.

*Results for missing values in education not displayed.*

In Model 2, educational attainment was added. Upper secondary education was found to have a significantly positive effect on correct knowledge compared with women with lower levels of education.

In Model 3, the indicators for religious family planning and gender role attitudes were added as explanatory variables. Note that the members of each migrant group differed significantly from women in the majority group regarding religiosity. Whereas only approximately 12% of non-migrants said that they followed religious regulations in family planning and birth control, the respective proportions among the migrant groups ranged from approximately 26% among women from the CIS countries to approximately 81% among women from Turkey. The proportion of women who perceive having children as an ideal was much higher in the migrant groups compared with the majority group (see Table 1). In the multivariate model (Model 3), religiosity was found to have a negative effect on fertility knowledge, but gender role attitudes had no additional significant effect in the multivariate model. Remarkably, the variables on education and religiosity mediated the main effect of group belonging to a small extent (i.e. the effect size of the origin group variable decreased). However, the finding of significantly lower knowledge among women belonging to most of the migrant groups in the study sample remained, indicated by negative average marginal effects.

### Multivariate findings by educational attainment

In order to test the role of social characteristics further, separate models by educational attainment were used, distinguishing between women with upper secondary education (or higher) and those who had less than upper secondary education (Table 3). Separate models by educational attainment were used in order to consider that the sociodemographic composition of the two educational groups may have varied. In separate models, the average effects of the explanatory variables were estimated within each group, whereas in joint models with, say, an interaction term, the mean effects of each variable would be calculated across the entire sample. In Models A1 and B1, the same sociodemographic indicators as in Table 2 were used as explanatory variables; in Models A2 and B2, cultural indicators were added.

**Table 3:** Multivariate results on knowledge on age-related fertility decline, by educational group.

School education	Below upper secondary						Upper secondary					
	Model A0		Model A1		Model A2		Model B0		Model B1		Model B2	
*Origin group (Ref = Germany)*												
Balkan countries	−0.10		−0.10		−0.07		−0.16	°	−0.13		−0.09	
Turkey	−0.18	*	−0.20	**	−0.15	*	−0.44	***	−0.42	***	−0.28	**
Poland	−0.13	**	−0.15	*	−0.11		−0.35	***	−0.33	***	−0.25	***
CIS countries	−0.14	*	−0.16	**	−0.13	*	−0.33	***	−0.31	***	−0.27	***
*Family planning conforming to religious regulations (Ref=*‘*disagree*’*/*‘*neither nor*’*)*												
‘(Completely) agree’					−0.06						−0.21	***
*n*	*584*		*584*		*584*		*378*		*378*		*378*	

*Source: NeWiRe survey 2014/2015.*

*Note: Binary logistic regression, dependent variable: 1 = correct, 0 = not correct;* °*P ≤ .1;* *P ≤ .05; **P ≤ .01; ***P ≤ .001.

*M1 controlled for age, union status, parenthood, wish to have more children, ART treatment experience;*

*M2 additionally controlled for gender role attitudes.*

First, this study found a minority-group disadvantage in both groups of formal education. Women of all four migrant groups had lower levels of knowledge, and this was significant at *P* ≤ 0.05 (Models A1 and B1), except for women from Balkan countries. This is remarkable because the size of the two educational subsamples was rather small and the confidence intervals were rather large. Therefore, the effect of the origin group on ARFD knowledge can be considered robust. Second, the effect size indicates that the gap in knowledge levels between the migrant and majority groups was higher in the higher educated group than in the lower-educated group.

Third, religiosity was found to work differently in the educational groups. In the lower-educated group, religiosity had a slightly negative, but not significant, impact on knowledge, and only explained a small proportion of the differences between the origin groups (Model A2). In contrast, in women with upper secondary education or higher, those who followed religious regulations in family planning had significantly lower levels of knowledge about fertility. In this educational group, religiosity contributed to explaining the differences between the origin groups to a larger extent than in the other educational group (Model B2). Finally, the reader should note that the overall pattern of group differences remained: women from Turkey, Poland and the CIS countries had lower likelihoods of correct knowledge about fertility than women from the Balkan region and German non-migrants, regardless of their educational level.

## Discussion

This paper aimed to explore whether migrant groups in Germany are at risk of reproductive marginalization. Data representative of the general population were used to investigate whether members of several migrant groups differed in their knowledge about ARFD compared with women in the majority group. To the best of the authors’ knowledge, this is the first study to target female migrants in Europe in a non-patient, and thus non-selected, sample and examine their awareness about fertility.

With respect to the working hypotheses, it was concluded that immigrant women in Germany have lower levels of fertility knowledge, supporting the working hypothesis of differences between the migrant and majority groups. Thus, the result suggests that the differences found between individuals living in developed and less-developed countries, such as Turkey (Bunting et al., 2013), continue after migration. This highlights the importance of the socialization context and early education. The gap in knowledge levels mainly occurred in first-generation migrants, and only carried over partially to second-generation migrants. Thus, the results deliver only partial evidence for the migrant generation/assimilation hypothesis. Similarly, only partial support was found for the compositional hypothesis; the third variables could not explain the differences between the majority and migrant groups.

More importantly, when testing the role of stratification by education, the effect size indicated that the gap in knowledge levels between migrant groups and the majority group was even greater in higher-educated women than in lower-educated women. This result suggests that female migrants do not use their higher education in fertility knowledge to the same extent as women in the majority group. Hence, it was concluded that higher education is not as beneficial for migrant women’s awareness about fertility as it is for women in the majority group. The adherence of female migrants to religious rules hampers educational gain somewhat. Women from Turkey, Poland and the CIS countries had lower likelihoods of correct knowledge about fertility than women from the Balkan region and German non-migrants, regardless of their educational level. This finding suggests that the lower level of fertility knowledge is not the result of socio-economic differences, and thus rejects the working hypothesis. Rather, it is indicative of a minority-group disadvantage.

Why does the significant minority-group disadvantage remain? The first explanation is cultural factors. Geographical variation in family formation patterns may play a large role. Migrants’ countries of origin differ from Germany in the sense that Germany has undergone developments known as the ‘second demographic transition’ (van de Kaa, 1987) far more thoroughly, with decoupling of marriage and childbearing. In Turkey, Balkan countries, Poland and CIS countries, family demographics remain characterized by the strong link between sexual activity, marriage and childbearing. Public discourses as well as research on awareness about fertility and reproductive health are more concerned with starting sexual activity and contraceptive use than advanced maternal age [e.g. Warzecha et al. (2019) for Poland]. Thus, sexual education and prevailing norms emphasize the importance of the ‘right’ conditions for childbearing. They do not explicitly target the age for childbearing, but set an indirect lower age norm by promoting the ideal of virginity at marriage. Such traditional family formation norms are de-facto implicit fertility age norms [Mynarska (2010) for Poland], although people may be unaware of this. The public omission of upper age norms for fertility is similar in many countries, and policy attempts to draw greater attention to the risks of advanced maternal age are highly controversial (Pedro et al., 2018), especially because young adults, and women in particular, are confronted with competing age norms in modernization processes. Educational expansion and growing female labour force participation, gender and family policies are associated with fertility postponement. The age at (first) motherhood has been rising more rapidly in Germany, and is higher than the age at (first) motherhood in these migrants’ regions of origin. In other words, the mean age at first birth in Germany today falls in the category with ‘slight ARFD’ (Frejka and Sardon, 2006). Postponement processes also occur in the regions of origin of the migrants in the study sample, albeit on a lower level. Fertility knowledge was shown to be lower in countries with rather early childbearing ages because most childbearing occurs before ARFD starts, so advanced maternal age may not emerge as a quantitatively important risk factor for infertility. Therefore, it is likely that the respondents in the study sample were not aware of the ARFD effect because of a lack of such experience in their country of origin and/or their migrant community.

A second possible explanation for the migrant disadvantage in fertility knowledge may lie in religiosity, which is higher in the migrant groups than in the majority population in Germany. Previous research on religious minorities has focused largely on Muslims, and posed the question whether this particular religion may work as a ‘bridge or barrier’ to migrant integration (Foner and Alba, 2008), with issues around gender equality and family patterns appearing as central social problems (Koopmans, 2015). The study survey also included women with Christian affiliations. Religiosity was found to be associated with lower fertility knowledge for Muslims as well as Christians. A specific indicator was used to proxy religiosity (i.e. the application of religious prescriptions in respondents’ own family planning). The proportion of respondents belonging to a religious community is, however, larger than this ‘religion’ proxy; some women may not practice family planning at the time of the survey for several reasons while still being religious overall. Moreover, some of the non-affiliated may still feel religious in a wider sense, but not follow religious doctrines in this specific area. Therefore, the extent of religiosity as such, as well as its impact on levels of awareness about fertility, may be underestimated, especially in contexts with generally higher religiosity, such as the examined migrant communities. Miron-Shatz et al. (2021) showed that individuals (female patients in reproductive treatment in Israel) may not lack information, but may choose to ignore it and instead overestimate their own success rates. The authors assume that a strong religious belief and adherence to religious prescriptions may support such cognitive dissonance between general information and own situation. People may think that religious and social criteria are the essential prerequisites to having a child; they may simply not care about (higher) age as a risk factor. In this sense, the title of the paper by Miron-Shatz, quoting a study participant – ‘Luckily, I don’t believe in statistics’ – may also apply to the present context and read literally as the – more or less – conscious replacement of statistical knowledge and comprehension with faith. There may also be different explanations for infertility within minority communities (Culley and Hudson, 2009).

The migrant disadvantage found may partially indicate marginalization. The explanations above do not seem to cover the whole story of the minority-group disadvantage in fertility knowledge, because the minority–majority gap persists even after controlling for education and religiosity, and also when examining each educational group separately, and does so with rather high statistical significance. Hence, these results may also be indicative of a marginalization effect among migrant groups in Germany, similar to the marginalization observed among ethnic minorities in the USA. Unlike in the USA, very little research has been undertaken on migrant groups or ethnic minorities in Europe that can be used to support the present hypotheses and explanations of the findings. The scarce evidence, however, suggests that migrant group members may encounter stereotyping, devaluation and discrimination in reproductive health practice. This may also include insufficient information on fertility and infertility risks, especially if healthcare professionals adhere to the stereotyping of hyperfertile and/or early fertile immigrant women. Although it may be a statistical fact that the average age at (first) birth is lower in migrant groups, and infertility may be less of a large-scale quantitative problem in these groups than in respective majority populations, their treatment experiences and discourses about help-seeking in reproductive health medicine nonetheless tell a story of belonging (or not) to a society. Greil et al. (2011) summed this up in the question, ‘Who deserves to become a parent or to have another child?’ Especially in cultural frames where the birth rate is high and where childlessness is rare, infertility and childlessness may have greater social consequences and may cause greater personal suffering, or even stigma and exclusion, for religious people and people in collectivistic cultures in which family is the main source of pride (Culley et al., 2009b, Culley and Hudson, 2009). Previous research on social norms and personal attitudes towards the use of ART demonstrated that migrants and religious women in Germany are more in favour of using reproductive medicine than non-migrants [Haug and Milewski, 2018, Milewski and Haug, 2020 (using the same data source)]. This shows that motherhood and having children remains an ideal for migrant women in Germany to a larger extent than for the majority population. Future research should investigate the role that reproductive healthcare services play in providing information on fertility risk factors, such as advanced age, to migrants. Previous research suggests that fertility postponement is also increasing in migrant and ethnic minority groups, resulting from women participating in higher education and aspiring to participate in the labour force (Culley and Hudson, 2009). It may also be worth investigating how discourses in the respective communities will develop and possibly affect fertility knowledge.

### Reflections on material and methods

Overall, the findings of this study suggest that women’s awareness about fertility, measured as knowledge about ARFD, is inadequate in Germany. In order to evaluate the generalizability of the findings from the NeWiRe pilot project, the results were compared with previous research. For better comparability, the following refers only to the respondents in the majority group from the study sample as other studies examined mainstream populations alone. Despite the low general knowledge level, respondents in the present study fared better than those in the study by Stoebel-Richter et al. (2012), which was the basis for the NeWiRe survey. Stoebel-Richter et al. found that only approximately 3% of women answered ‘at age 25’, which they considered ‘correct’ (corresponding to 14% in the present study), and approximately 11% answered ‘at age 30’, which also would have counted as ‘correct’ in the present study (corresponding to 29% in the present study). However, the percentage of those who said that fertility starts to decline at ‘age 40+’ or ‘during menopause’ was only slightly lower in the present sample (27%) than in the study by Stoebel-Richter et al. (2012; 30%).

On the one hand, the compositional differences of the survey samples with respect to education may have caused the differences in knowledge; the present sample had a much higher proportion of higher-educated participants [47% versus 23% in Stoebel-Richter et al. (2012) with at least upper secondary education]. Comparing the educational attainment in the present sample with other data suggests that the present data captured the educational structure of German non-migrants rather well (OECD, 2019). On the other hand, in an international comparison, the majority population respondents were not more knowledgeable than those in other countries [Garcia et al. (2018) and Hammarberg et al. (2013) for a sample of the general population in Australia; Vassard et al. (2016) for Denmark and the UK). Here, the issue of comparability may play a role. The answers in the present questionnaire started at ‘age 25+’, those in Vassard et al. (2016) started at ‘age 20+’, and those in Hammarberg et al. (2016) started with age ‘<30’. Moreover, the present study and the study by Hammarberg et al. (2016) included ‘during menopause’ as a possible answer category, whereas the study by Vassard et al. (2016) did not offer such an option. Hence, the chances of this answer were equal to 0 by default, and the respondents were prompted to an answer referring to age. These similarities and differences suggest that measured levels of awareness about fertility are also driven by the socio-economic composition of the samples, as well as by survey design (Garcia et al., 2018).

In this study, the dependent variable only examined advanced maternal age as a risk factor for infertility. Whereas age is non-modifiable, there are some other risk factors for which timely intervention or prevention could help. Therefore, it may be useful to ask further questions on fertility knowledge in future studies on migrant groups, especially groups coming from countries where education on sexual behaviour and reproductive health is highly gendered, and surrounded by myths and stigma. The 13-item Cardiff Fertility Knowledge Scale, which includes modifiable risk factors such as obesity, sexually transmitted infections, smoking and the absence of menstruation, could be used (Bunting et al., 2013). Future research may also include more questions on knowledge about assisted conception, such as success rates, because members of minority groups may be more open to using ART than the majority group in Germany (Haug and Milewski, 2018). Their knowledge about ART, however, has not been studied to date.

## Conclusions

Migrant groups appear to be under-represented – thus, marginalized – in research on awareness about fertility. If certain social groups are omitted from analyses, their potential needs become ignored. Such exclusion does not only occur in the area of reproductive health. It is the most obvious form of institutional racism towards minority populations (Atkin, 2009), and constitutes a commonality between migrant groups in Europe and ethnic minorities in Northern America. This study suggests that future research should actively include and examine minorities in analyses on infertility and reproductive medicine, at least where omission is based on the assumption that certain social groups have no disadvantages in family planning or are not at risk of infertility and age-related complications. This study illustrated that such assumptions may not hold for migrant groups in Germany. Moreover, the analyses suggest that in addition to women from predominantly Muslim countries, Eastern Europeans with rather Christian traditions may also be at risk of reproductive disadvantage, while members of both migrant groups adhere to motherhood as a gender role ideal more stringently than the majority population.

## Declaration

The author reports no financial or commercial conflicts of interest.
